# Supplementary material for: Consistent RNA sequencing contamination in GTEx and other data sets
Source: Nat Commun. 2020 Apr 22;11:1933. doi: 10.1038/s41467-020-15821-9 (PMC7176728; doi:10.1038/s41467-020-15821-9)
Supplement: Supplementary file 1 — Supplementary Information [file 41467_2020_15821_MOESM1_ESM.pdf]

Consistent RNA Sequencing Contamination in GTEx and Other Datasets

Tim O. Nieuwenhuis et al.

Supplementary Information

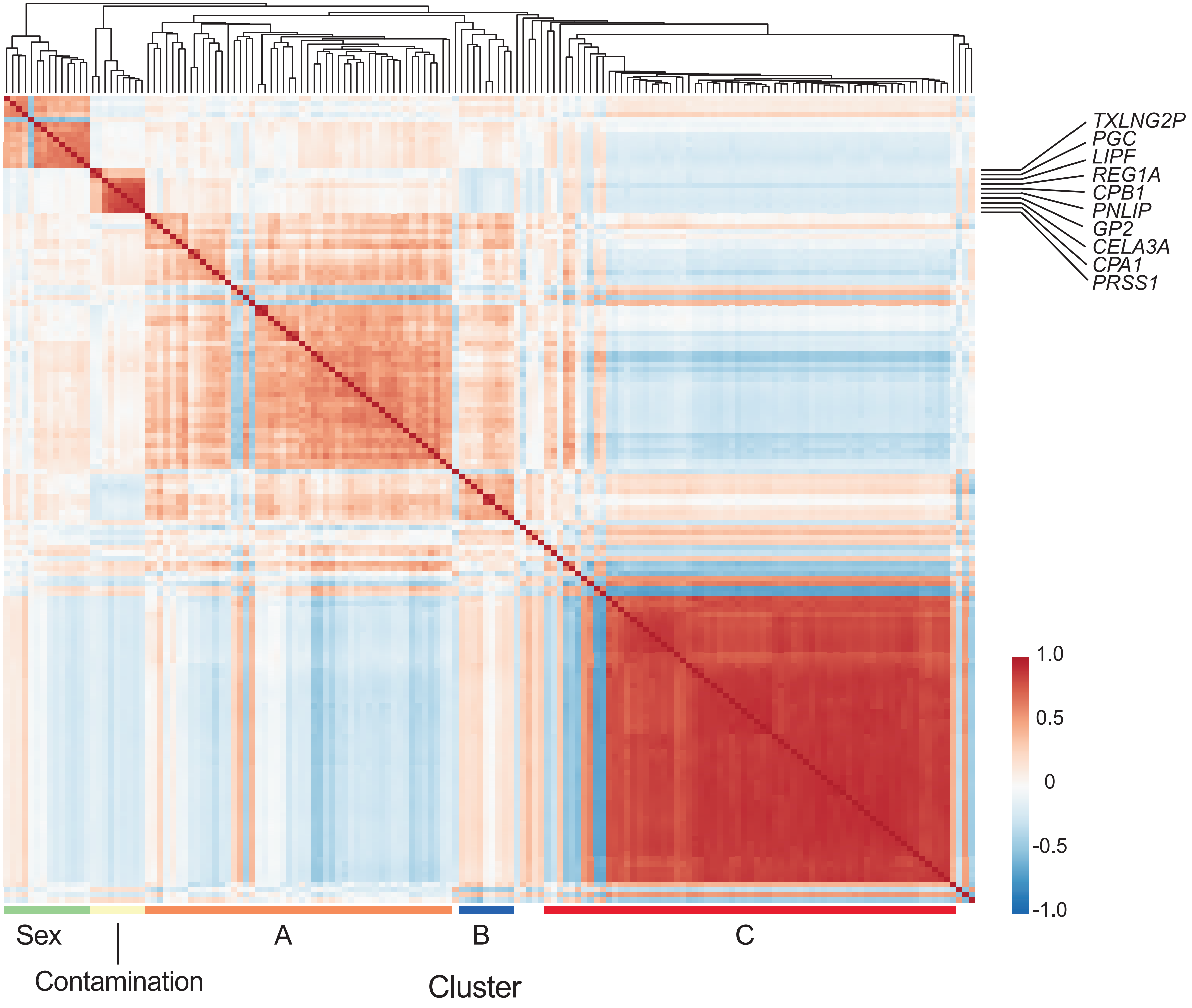

TXLNG2P
PGC
LIPF
REG1A
CPB1
PNLIP
GP2
CELA3A
CPA1
PRSS1
1.0
0.5
0
-0.5
-1.0
Sex
Contamination
A
B
C
Cluster

Supplementary Figure 1. **A correlation heatmap of the highly variable gene clusters in 343 transformed fibroblast samples.** Blue to red scale shows Kendall's tau correlation from -1 to 1. Genes within the contamination cluster are given. The etiology of clusters A and B are unknown. Cluster C represents co-variable genes related to mitosis.

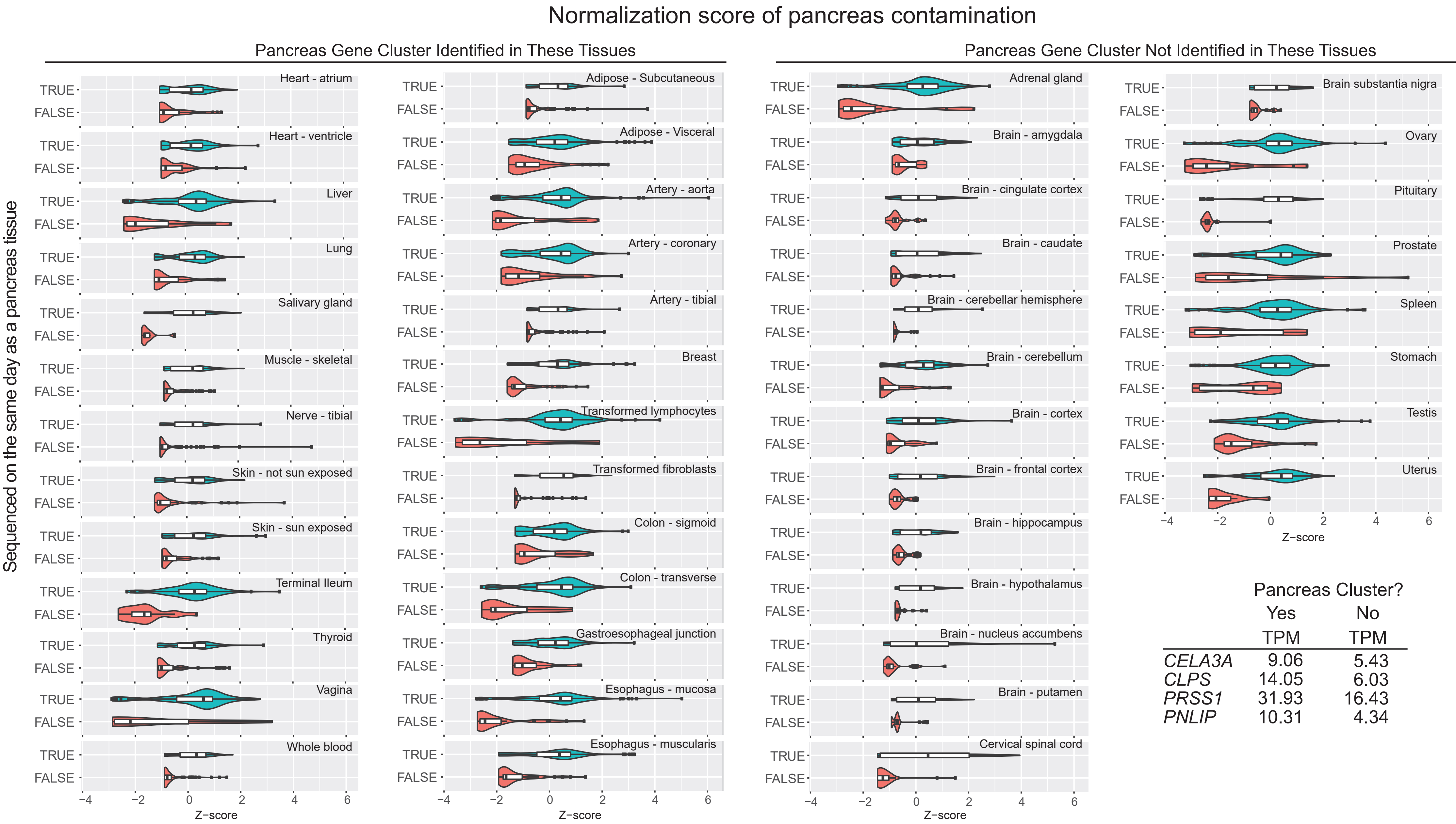
Normalization score of pancreas contamination
Pancreas Gene Cluster Identified in These Tissues
Pancreas Gene Cluster Not Identified in These Tissues
Sequenced on the same day as a pancreas tissue
TRUE
FALSE
Z−score
Heart - atrium
Heart - ventricle
Liver
Lung
Salivary gland
Muscle - skeletal
Nerve - tibial
Skin - not sun exposed
Skin - sun exposed
Terminal Ileum
Thyroid
Vagina
Whole blood
Adipose - Subcutaneous
Adipose - Visceral
Artery - aorta
Artery - coronary
Artery - tibial
Breast
Transformed lymphocytes
Transformed fibroblasts
Colon - sigmoid
Colon - transverse
Gastroesophageal junction
Esophagus - mucosa
Esophagus - muscularis
Adrenal gland
Brain - amygdala
Brain - cingulate cortex
Brain - caudate
Brain - cerebellar hemisphere
Brain - cerebellum
Brain - cortex
Brain - frontal cortex
Brain - hippocampus
Brain - hypothalamus
Brain - nucleus accumbens
Brain - putamen
Cervical spinal cord
Brain substantia nigra
Ovary
Pituitary
Prostate
Spleen
Stomach
Testis
Uterus
Pancreas Cluster?
Yes No
TPM TPM
CELA3A 9.06 5.43
CLPS 14.05 6.03
PRSS1 31.93 16.43
PNLIP 10.31 4.34

Supplementary Figure 2. Violin plots representing the pancreatic gene (*CELA3A*, *CLPS*, *PRSS1*, *PNLIP*) normalization scores of samples sequenced the same day as a pancreas sample and those that were not. The bottom right corner shows the median TPM of pancreas genes in non-pancreas tissues. These were divided base on the tissue having all four genes in their contamination cluster (N=26) or not (N=21). The solid line in all boxplots represents the median of the data, while the lower and upper hinges correspond to the 25th percentile and 75th percentile respectively. The whiskers represent the interquartile range × 1.5, and any outliers beyond the whiskers are represented as dots

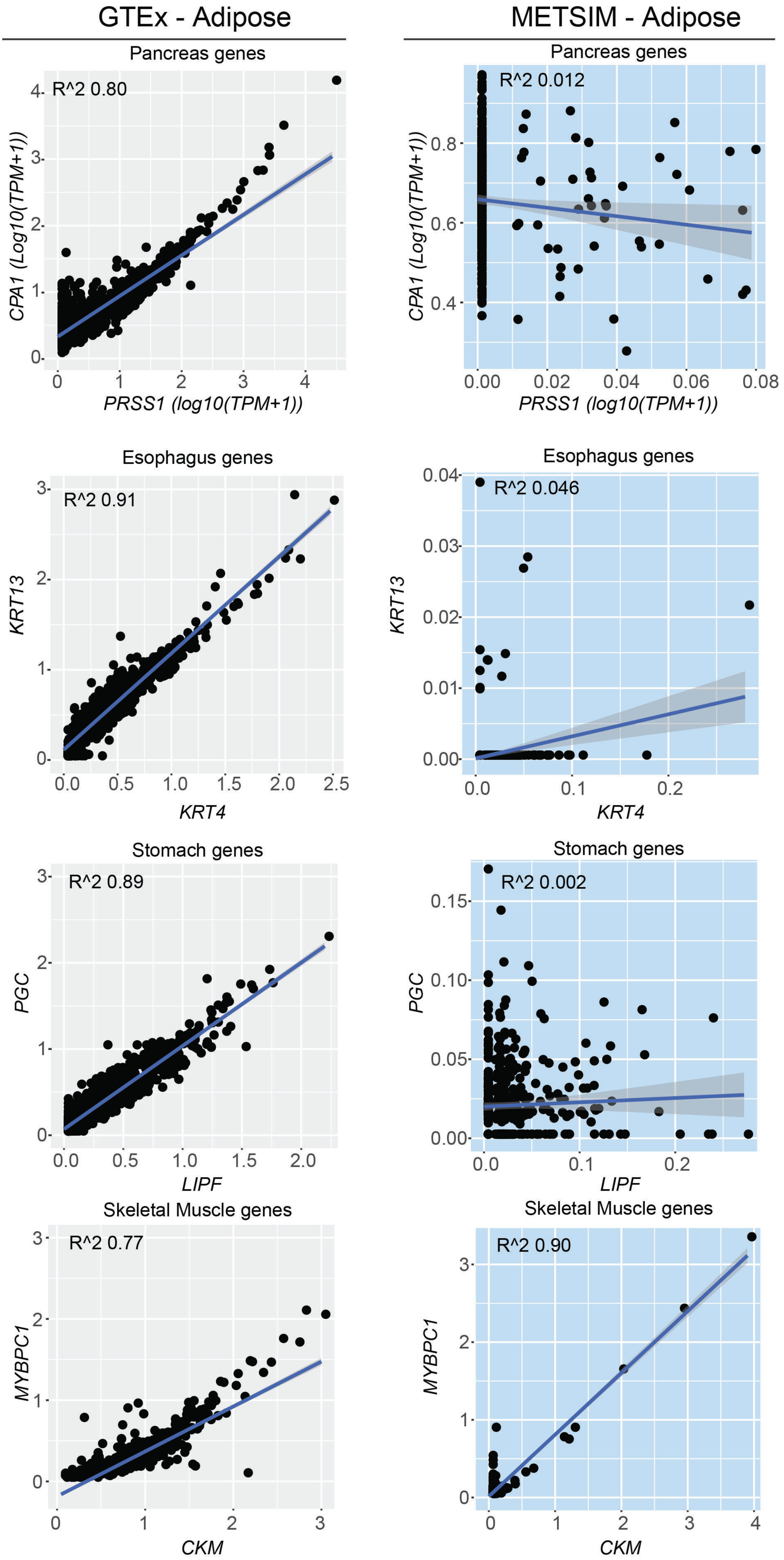
GTEx - Adipose
METSIM - Adipose
Pancreas genes
R^2 0.80
CPA1 (Log10(TPM+1))
PRSS1 (log10(TPM+1))
Pancreas genes
R^2 0.012
CPA1 (Log10(TPM+1))
PRSS1 (log10(TPM+1))
Esophagus genes
R^2 0.91
KRT13
KRT4
Esophagus genes
R^2 0.046
KRT13
KRT4
Stomach genes
R^2 0.89
PGC
LIPF
Stomach genes
R^2 0.002
PGC
LIPF
Skeletal Muscle genes
R^2 0.77
MYBPC1
CKM
Skeletal Muscle genes
R^2 0.90
MYBPC1
CKM

Supplementary Figure 3. A scatterplot comparison of different contaminating genes between the GTEx adipose tissues (n = 797) and the adipose tissues harvested in the METSIM study (n = 434). We compared *CPA1* vs *PRSS1*, *KRT13* vs *KRT4*, *PGC* vs *LIPF*, and *MYBPC1* vs *CKM* as each of those gene pairs are highly expressed together in a contaminating tissue type. In GTEx the expression is consistently higher and has a stronger positive correlation compared to the METSIM study for three of those pairs because skeletal muscle can be captured in adipose harvesting, *MYBPC1* and *CKM* also showed strong correlation in METSIM as a result of a direct contamination.

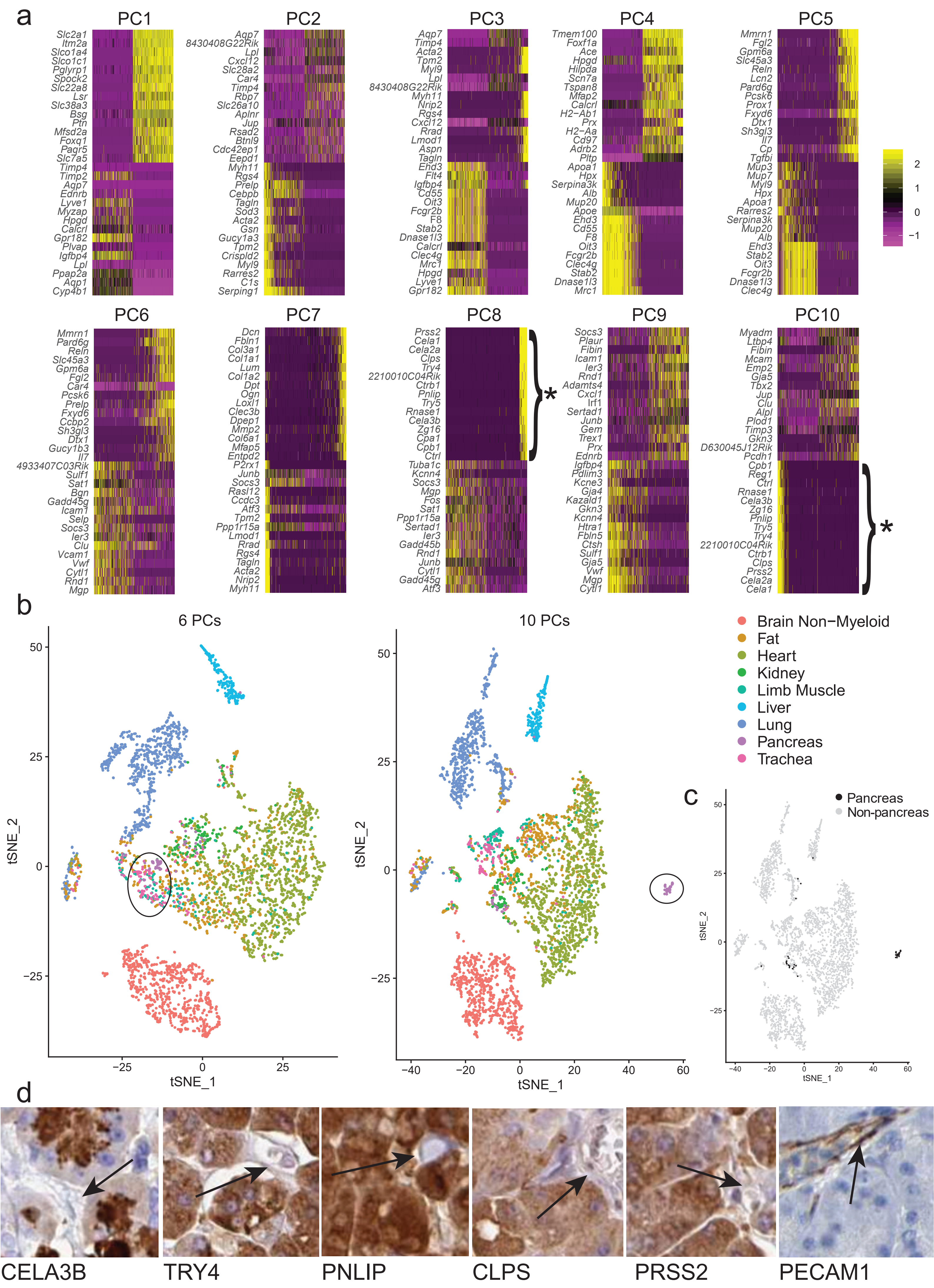
a
PC1
Slc2a1
Itm2a
Slco1a4
Slco1c1
Pglyrp1
Spock2
Slc22a8
Lsr
Slc38a3
Bsg
Ptn
Mfsd2a
Foxq1
Pagr5
Slc7a5
Timp4
Timp2
Aqp7
Ednrb
Lyve1
Myzap
Hpgd
Calcrl
Gpr182
Plvap
Igfbp4
Lpl
Ppap2a
Aqp1
Cyp4b1
PC2
Aqp7
8430408G22Rik
Lpl
Cxcl12
Slc28a2
Car4
Timp4
Rbp7
Slc26a10
Aplnr
Jup
Rsad2
Btnl9
Cdc42ep1
Eepd1
Myh11
Rgs4
Prelp
Cebpb
Tagln
Sod3
Acta2
Gsn
Gucy1a3
Tpm2
Crispld2
Myl9
Rarres2
C1s
Serping1
PC3
Aqp7
Timp4
Acta2
Tpm2
Myl9
Lpl
8430408G22Rik
Myh11
Nrip2
Rgs4
Cxcl12
Rrad
Lmod1
Aspn
Tagln
Ehd3
Flt4
Igfbp4
Cd55
Oit3
Fcgr2b
F8
Stab2
Dnase1l3
Calcrl
Clec4g
Mrc1
Hpgd
Lyve1
Gpr182
PC4
Tmem100
Foxf1a
Ace
Hpgd
Hilpda
Scn7a
Tspan8
Mfap2
Calcrl
H2−Ab1
Prx
H2−Aa
Cd97
Adrb2
Pltp
Apoa1
Hpx
Serpina3k
Alb
Mup20
Apoe
Ehd3
Cd55
F8
Oit3
Fcgr2b
Clec4g
Stab2
Dnase1l3
Mrc1
PC5
Mmrn1
Fgl2
Gpm6a
Slc45a3
Reln
Lcn2
Pard6g
Pcsk6
Prox1
Fxyd6
Dtx1
Sh3gl3
Il7
Cp
Tgfbi
Mup3
Mup7
Myl9
Hpx
Apoa1
Rarres2
Serpina3k
Mup20
Alb
Ehd3
Stab2
Oit3
Fcgr2b
Dnase1l3
Clec4g
2
1
0
−1
PC6
Mmrn1
Pard6g
Reln
Slc45a3
Gpm6a
Fgl2
Car4
Pcsk6
Prelp
Fxyd6
Ccbp2
Sh3gl3
Dtx1
Gucy1b3
Il7
4933407C03Rik
Sulf1
Sat1
Bgn
Gadd45g
Icam1
Selp
Socs3
Ier3
Clu
Vcam1
Vwf
Cytl1
Rnd1
Mgp
PC7
Dcn
Fbln1
Col3a1
Col1a1
Lum
Col1a2
Dpt
Ogn
Loxl1
Clec3b
Dpep1
Mmp2
Col6a1
Mfap5
Entpd2
P2rx1
Junb
Socs3
Rasl12
Ccdc3
Atf3
Tpm2
Ppp1r15a
Lmod1
Rrad
Rgs4
Tagln
Acta2
Nrip2
Myh11
PC8
Prss2
Cela1
Cela2a
Clps
Try4
2210010C04Rik
Ctrb1
Pnlip
Try5
Rnase1
Cela3b
Zg16
Cpa1
Cpb1
Ctrl
*
Tuba1c
Kcnn4
Socs3
Mgp
Fos
Sat1
Ppp1r15a
Sertad1
Ier3
Gadd45b
Rnd1
Junb
Cytl1
Gadd45g
Atf3
PC9
Socs3
Plaur
Fibin
Icam1
Ier3
Rnd1
Adamts4
Cxcl1
Irf1
Sertad1
Junb
Gem
Trex1
Prx
Ednrb
Igfbp4
Pdlim3
Kcne3
Gja4
Kazald1
Gkn3
Kcnn4
Htra1
Fbln5
Ctsh
Sulf1
Gja5
Vwf
Mgp
Cytl1
PC10
Myadm
Ltbp4
Fibin
Mcam
Emp2
Gja5
Tbx2
Jup
Clu
Alpl
Plod1
Timp3
Gkn3
D630045J12Rik
Pcdh1
Cpb1
Reg1
Ctrl
Rnase1
Cela3b
Zg16
Pnlip
Try5
Try4
2210010C04Rik
Ctrb1
Clps
Prss2
Cela2a
Cela1
*
b
6 PCs
10 PCs
tSNE_2
tSNE_1
50
25
0
−25
−25
0
25
−40
−20
0
20
40
60
Brain Non−Myeloid
Fat
Heart
Kidney
Limb Muscle
Liver
Lung
Pancreas
Trachea
c
Pancreas
Non-pancreas
tSNE_2
tSNE_1
d
CELA3B
TRY4
PNLIP
CLPS
PRSS2
PECAM1

Supplementary Figure 4. Pancreatic acinar cell contamination drives endothelial cell diversity in Tabula Muris. a The major genes of the top 10 principal components (PCs) for nine groups of endothelial cells. * represents contaminating acinar cell expression. **b** tSNE plots based on 6 or 10 PCs demonstrate a large deviation of pancreas endothelial cells from a shared cluster to a unique cluster based on additional PCs. **c** In this black and white representation, a subset of pancreas cells are noted to move. **d** Representative images (HPA) of several highly-expressed acinar proteins that do not mark endothelial cells (black arrows) and PECAM1, a known endothelial cell marker.
