# Supplementary material for: Consistent RNA sequencing contamination in GTEx and other data sets
Source: Nat Commun. 2020 Apr 22;11:1933. doi: 10.1038/s41467-020-15821-9 (PMC7176728; doi:10.1038/s41467-020-15821-9)
Supplement: Supplementary file 3 — Reporting Summary [file 41467_2020_15821_MOESM3_ESM.pdf]

nature research

Corresponding author(s): Halushka, Marc

Last updated by author(s): Feb 25, 2020

Reporting Summary

Nature Research wishes to improve the reproducibility of the work that we publish. This form provides structure for consistency and transparency in reporting. For further information on Nature Research policies, see Authors & Referees and the Editorial Policy Checklist.

Statistics

For all statistical analyses, confirm that the following items are present in the figure legend, table legend, main text, or Methods section.

n/a	Confirmed	
☐	☒	The exact sample size (n) for each experimental group/condition, given as a discrete number and unit of measurement
☒	☐	A statement on whether measurements were taken from distinct samples or whether the same sample was measured repeatedly
☐	☒	The statistical test(s) used AND whether they are one- or two-sided<br>*Only common tests should be described solely by name; describe more complex techniques in the Methods section.*
☐	☒	A description of all covariates tested
☐	☒	A description of any assumptions or corrections, such as tests of normality and adjustment for multiple comparisons
☐	☒	A full description of the statistical parameters including central tendency (e.g. means) or other basic estimates (e.g. regression coefficient) AND variation (e.g. standard deviation) or associated estimates of uncertainty (e.g. confidence intervals)
☐	☒	For null hypothesis testing, the test statistic (e.g. F, t, r) with confidence intervals, effect sizes, degrees of freedom and P value noted<br>*Give P values as exact values whenever suitable.*
☒	☐	For Bayesian analysis, information on the choice of priors and Markov chain Monte Carlo settings
☒	☐	For hierarchical and complex designs, identification of the appropriate level for tests and full reporting of outcomes
☐	☒	Estimates of effect sizes (e.g. Cohen's d, Pearson's r), indicating how they were calculated

Our web collection on statistics for biologists contains articles on many of the points above.

Software and code

Policy information about availability of computer code

Data collection	All data used in this study was collected through dbGap (https://www.ncbi.nlm.nih.gov/gap/), GEO (https://www.ncbi.nlm.nih.gov/gds), recount2 (https://lcolladotor.github.io/project/recount2/) or Tabula Muris (https://figshare.com/articles/Robject_files_for_tissues_processed_by_Seurat/5821263).
Data analysis	We used R (3.6.1) and the following R packages: recount version 1.8.2, DESeq2 v1.22.1, Seurat v3.1.1. We used Python v3.6.2 and bam-readcount v0.8.0. We used the alignment tools HISAT2 v2.1.0, samtools v1.9, StringTie v1.3.4d and the Integrative Genome Viewer v2.4.12.

For manuscripts utilizing custom algorithms or software that are central to the research but not yet described in published literature, software must be made available to editors/reviewers. We strongly encourage code deposition in a community repository (e.g. GitHub). See the Nature Research guidelines for submitting code & software for further information.

Data

Policy information about availability of data

All manuscripts must include a data availability statement. This statement should provide the following information, where applicable:

- Accession codes, unique identifiers, or web links for publicly available datasets
- A list of figures that have associated raw data
- A description of any restrictions on data availability

The following accession codes were used to collect data: phs000424.v8.p2, ERP009437, SRP053101, SRP032833, SRP003611, ERP010889, GSE103905, GSE68229, GSE30611, SRP029880, ERP003613, GSE120795, GSE135134, GSE84133; GSE103322, GSE72056.
Tabula Muris data from: https://figshare.com/articles/Robject_files_for_tissues_processed_by_Seurat/5821263

Field-specific reporting

Please select the one below that is the best fit for your research. If you are not sure, read the appropriate sections before making your selection.

☒ Life sciences ☐ Behavioural & social sciences ☐ Ecological, evolutionary & environmental sciences

For a reference copy of the document with all sections, see nature.com/documents/nr-reporting-summary-flat.pdf

Life sciences study design

All studies must disclose on these points even when the disclosure is negative.

Sample size	Sample sizes were based on the available publicly-available data sets.
Data exclusions	GTEx tissues with <70 samples were excluded from the study.
Replication	Replication was performed by finding datasets similar to GTEx and demonstrating how the same type of highly-expressed organ-specific sequence could be found in inappropriate tissues.
Randomization	Randomization was not relevant to our study.
Blinding	Blinding was not relevant to our study.

Reporting for specific materials, systems and methods

We require information from authors about some types of materials, experimental systems and methods used in many studies. Here, indicate whether each material, system or method listed is relevant to your study. If you are not sure if a list item applies to your research, read the appropriate section before selecting a response.

Materials & experimental systems

n/a	Involved in the study	
☒	☐	Antibodies
☒	☐	Eukaryotic cell lines
☒	☐	Palaeontology
☒	☐	Animals and other organisms
☐	☒	Human research participants
☒	☐	Clinical data

Methods

n/a	Involved in the study	
☒	☐	ChIP-seq
☒	☐	Flow cytometry
☒	☐	MRI-based neuroimaging

Human research participants

Policy information about studies involving human research participants

Population characteristics	The main investigated population was the GTEx study. The GTEx portal and dbGAP provides all relevant population characteristics.
Recruitment	Recruitment was performed through GTEx and Organ Procurement Organizations (OPOs).
Ethics oversight	GTEx was approved by the NIH with appropriate ethics oversight.

Note that full information on the approval of the study protocol must also be provided in the manuscript.
