# Supplementary material for: Consistent RNA sequencing contamination in GTEx and other data sets
Source: Nat Commun. 2020 Apr 22;11:1933. doi: 10.1038/s41467-020-15821-9 (PMC7176728; doi:10.1038/s41467-020-15821-9)
Supplement: Supplementary file 4 — Description of Additional Supplementary Files [file 41467_2020_15821_MOESM4_ESM.pdf]

Description of Additional Supplementary Files

File Name: Supplementary Data 1

Description: Ratio of tissues sequenced on pancreas and non-pancreas days.

File Name: Supplementary Data 2

Description: Ratio of tissues sequenced on esophagus and non-esophagus days.

File Name: Supplementary Data 3

Description: The output of the pancreas gene contamination linear mixed model.

File Name: Supplementary Data 4

Description: The output of the esophagus mucosa gene contamination linear mixed model.

File Name: Supplementary Data 5

Description: A technical comparison of the GTEX1 fibroblast sample and its main contaminating GTEX2 esophagus sample.

File Name: Supplementary Data 6

Description: Ten RNA-seq experiments with two or more tissues/cells all demonstrating cross contamination between samples.
